# Determining effects of areca (betel) nut chewing in a prospective cohort of pregnant women in Madang Province, Papua New Guinea

**DOI:** 10.1186/s12884-015-0615-z

**Published:** 2015-08-19

**Authors:** Maria Ome-Kaius, Holger W Unger, Dupain Singirok, Regina A Wangnapi, Sarah Hanieh, Alexandra J Umbers, Julie Elizah, Peter Siba, Ivo Mueller, Stephen J Rogerson

**Affiliations:** Papua New Guinea Institute of Medical Research (PNG IMR), PO BOX 60, Goroka, Eastern Highlands Province 411 Papua New Guinea; Department of Medicine (Royal Melbourne Hospital), The University of Melbourne, Melbourne, Post Office Royal Hospital, Parkville, Melbourne, VIC 3050 Australia; Walter and Eliza Hall Institute of Medical Research (WEHI), 1G Royal Parade, 3052 Parkville, Australia; Barcelona Centre for International Health Research (CRESIB), Rosselo 132, 08036 Barcelona, Spain

## Abstract

**Background:**

Chewing areca nut (AN), also known as betel nut, is common in Asia and the South Pacific and the habit has been linked to a number of serious health problems including oral cancer. Use of AN in pregnancy has been associated with a reduction in mean birthweight in some studies, but this association and the relationship between AN chewing and other adverse pregnancy outcomes remain poorly understood.

**Methods:**

We assessed the impact of AN chewing on adverse outcomes including stillbirth, low birthweight (LBW, <2,500 g) and anaemia at delivery (haemoglobin <11.0 g/dL) in a longitudinal cohort of 2,700 pregnant women residing in rural lowland Papua New Guinea (PNG) from November 2009 until February 2013. Chewing habits and participant characteristics were evaluated at first antenatal visit and women were followed until delivery.

**Results:**

83.3 % [2249/2700] of pregnant women used AN, and most chewed on a daily basis (86.2 % [1939/2249]. Smoking and alcohol use was reported by 18.9 % (511/2700) and 5.0 % (135/2688) of women, respectively.

AN use was not associated with pregnancy loss or congenital abnormalities amongst women with a known pregnancy outcome (*n* = 2215). Analysis of 1769 birthweights did not demonstrate an association between AN and LBW (chewers: 13.7 % [200/1459] vs. non-chewers: 14.5 % [45/310], *P* = 0.87) or reduced mean birthweight (2957 g vs. 2966 g; *P* = 0.76). Women using AN were more likely to be anaemic (haemoglobin <11 g/dL) at delivery (75.2 % [998/1314] vs. 63.9 % [182/285], adjusted odds ratio [95 % CI]: 1.67 [1.27, 2.20], *P* < 0.001). Chewers more commonly had male babies than non-chewers (46.1 % [670/1455] vs. 39.8 % [123/309], *P* = 0.045).

**Conclusions:**

AN chewing may contribute to anaemia. Although not associated with other adverse pregnancy outcome in this cohort gestational AN use should be discouraged, given the potential adverse effects on haemoglobin and well-established long-term health risk including oral cancer. Future research evaluating the potential association of AN use and anaemia may be warranted.

**Trial registration:**

ClinicalTrials.gov NCT01136850 (06 April 2010).

## Background

Areca nut (AN), also known as betel nut, is the fruit of the *Areca catechu* Linn. palm tree. Across the globe, approximately 600 million people use AN as a masticatory drug, primarily in tropical and sub-tropical regions of South-East Asia and the South Pacific [1, 2]. The psycho-stimulatory effects of AN chewing are thought to be mediated by the alkaloid arecoline [3, 4]. Other alkaloids contained in AN, specifically guavacoline, guavacine, arecaidine, may also play a role [5]. After nicotine, alcohol and caffeine, arecoline from AN is the most widely used addictive substance globally [1].

AN has been associated with oral cancer as a result of exposure to carcinogenic derivatives of arecoline [5, 6]. Use also increases the risk of hypertension and arrhythmias [7, 8], possibly due to the effects of arecoline on the autonomic nervous system [3, 9], and has been linked to endocrine disease such as diabetes [10, 11]. These adverse effects have been demonstrated in a range of different geographical settings including Papua New Guinea (PNG) [8, 12], where widespread AN use has been reported [13–15].

AN use is not uncommon in pregnancy in most regions where it is consumed [15–17]. This raises concerns about its potentially detrimental effects on embryogenesis and fetal growth, and hence AN use could represent an important target for public health interventions to reduce the burden of low birthweight (LBW, birthweight <2500 g) and infant mortality [18, 19]. AN has been associated with reduction in birthweight in two pregnancy studies undertaken in PNG [15, 20]. Similarly, AN consumption was associated with reduced birthweight, reduced fetal length as well as LBW in research from Taiwan [16, 21, 22]. This research also identified an association between AN use and miscarriage, stillbirth, preterm delivery (PTD, <37 gestational weeks) and increased male-to-female ratio [16, 21, 22]. In contrast, two other studies [17, 23] were unable to demonstrate deleterious effects of AN use on pregnancy outcomes, birthweight and anaemia in pregnancy.

In addition to study design and sample size, explanations for the differences in the effect of AN on birthweight observed between studies may include variations in how ANs are prepared and chewed [2, 24]. In a number of settings betel nut is chewed together with tobacco, and associations with smokeless tobacco use and adverse birth outcomes are well documented [21, 25]. Furthermore, it has been demonstrated that availability and uptake of arecoline depends on the degree of maturity of the nuts at the time of consumption, the method of preparation, the contents of the ‘betel quid’, and whether the quid is swallowed or spat out [5]. Betel quid describes the combination of AN with ingredients such as *Piper betel* leaves, slaked lime (calcium hydroxide), tobacco, and sweet spices for consumption [1, 2, 26]. In PNG, AN, locally known as ‘buai’*,* is usually chewed in combination with the *Piper betel* fruit (known as ‘daka’) and lime (known as ‘kambang’), the latter serving as a catalyst [12, 15]. Unlike other regions, tobacco is not chewed in PNG [27]. AN chewing is equally prevalent among men and women in the adult PNG population [13, 28].

This study assessed the effects of AN chewing on pregnancy outcomes, birthweight, and anaemia in a large cohort of pregnant women enrolled before or at 26 gestational weeks and followed until delivery.

## Methods

### Study design, setting and participants

This prospective cohort study of pregnant women was conducted at nine health facilities in Madang Province on the North East Coast of PNG. Women were enrolled between November 2009 and August 2012, and follow-up was completed in February 2013. Participants were co-enrolled in a clinical trial that evaluated the impact of intermittent preventive treatment in pregnancy (IPTp) with the combination of sulphadoxine-pyrimethamine and azithromycin (SPAZ) on LBW [29]. The study area was previously reported to have a high prevalence of AN use [15], malaria and anaemia [30, 31], and LBW [15]. Antenatal HIV prevalence at Madang’s Modilon hospital was 1.1 % (34/3108) (2009–2012, unpublished audit data).

Women attending their first antenatal care visit were provided with study information and invited to participate. Interested women were screened for eligibility and were excluded if they had i) symphysis-pubis fundal height >26 cm, ii) haemoglobin (Hb) <6 g/dL and were symptomatic as a result of anaemia, iii) previous adverse reaction to study medications, iv) permanent disability preventing participation, v) known multiple pregnancy, vi) unavailable for follow-up, viii) not pregnant, ix) residing out with study area, or x) unable to report on AN use. Routine clinical assessments were performed and socio-demographic and clinical data were collected at enrolment using standardized case report forms. Participants were screened for syphilis and anaemia. HIV counseling and testing was provided at participating health centers but information on serostatus was not collected as part of this study. Women presenting with signs and symptoms of malaria or Hb <9 g/dL (HemoCue, Ängelholm, Sweden; accuracy of 0.1 g/dL) were tested using a malaria rapid diagnostic test (CareStart™ Pf/Pv combo test, AccessBio, USA). Malaria and anaemia were treated with artemether/lumefantrine (2^nd^ & 3^rd^ trimester) or quinine (first trimester), and women with Hb <9 g/dL were provided with iron/folate supplements (Fefol 2 tabs once daily) and albendazole (400 mg stat), as per national guidelines [32]. In addition, peripheral blood smears were prepared at enrolment and subsequent IPTp visits (2), at delivery, and during passive case detection visits: smears were used for the evaluation of the malaria burden only. Participants were scheduled for an obstetric ultrasound examination (Logiqbook XP, General Electric Medical Systems, UK) to date their pregnancy: we were unable to offer scans to all women for operational reasons.

Study participants were followed up until delivery when information on the outcome of pregnancy and birthweight was collected.

### Measures

The primary outcome measures of the study were LBW and mean birthweight. Secondary outcomes included anaemia and mean arterial pressure at delivery, PTD, neonatal length, miscarriage, stillbirth, congenital abnormalities, and infant sex.

Maternal AN use (exposure) was evaluated at enrolment and based on information reported by participants. We enquired whether women chewed or not, and subsequently evaluated the frequency of chewing, with women estimating their average number of ANs chewed per day. Serum arecoline levels were not measured. Amongst chewers, we estimated the number of ANs chewed per day as ‘less than one nut per day’, ‘1-2 nuts per day’, ‘3–5 nuts per day’ and ‘more than 6 nuts per day’, we assumed chewing patterns as reported at baseline would continue during pregnancy [15, 20].

Potential confounders/effect modifiers were measured and included smoking, gravidity, malaria, alcohol use, ethnicity/regional origin, residence, literacy level (defined as no education or education less than grade 3), income (participant and partner), maternal nutritional status, bed net use, maternal height, frequency of antenatal clinic visits, receipt of IPTp, infant gender and timing of birthweight/haemoglobin measurement.

Smoking and alcohol consumption during the index pregnancy were evaluated at enrolment only. Questionnaires did not quantify intake or characterize types of tobacco smoked and alcohol products consumed.

Research nurses measured birthweight using an electronic infant scale (Cupid 1, Charder Medical, Taiwan, precision 10 g); only birthweights measured within 24 h of delivery were used in analyses.

Miscarriage was defined as a delivery <22 weeks gestation or birthweight <500 g [29]. Neonatal anthropometry included head circumference, length and abdominal circumference, and newborns were examined for congenital malformations.

PTD was defined as a delivery before 37 gestational weeks [33]. Last menstrual period and Ballard scores were highly unreliable in accurately estimating gestational age in our cohort (data not shown). Therefore, the potential impact of AN use on PTD was only assessed amongst women who had ultrasound-based pregnancy dating.

At delivery, Hb levels and blood pressure (Automatic Blood Pressure Monitor, Omron, Australia) were measured. Anaemia was defined as an Hb <11 g/dL [34], and mean arterial pressure (diastolic pressure plus one third of pulse pressure) was calculated.

Malarial infection was defined as detection of parasitaemia in peripheral venous blood by light microscopy. Blood smears were examined as previously described [29].

Clinical staff assessing primary and secondary outcomes were not told of women’s chewing status as reported at enrolment; however complete blinding was impossible given the effect of frequent chewing on dental health.

### Statistical analysis

Data was doubled-entered into a study database (FoxPro 9.0, Microsoft, USA) and analysed using Stata 12.0 (StataCorp, USA).

Continuous parametric data was described as mean ± standard deviation (SD), continuous nonparametric data as median and interquartile range (IQR) and categorical data as % (n). When comparisons of variables across all five groups remained significant after Bonferroni adjustments, pairwise comparisons of the different chewing level groups against non-chewers were performed using the two-sided student’s *t*-test for parametric data, the Mann Whitney-*U* test for nonparametric data, and the Chi-squared or Fisher’s exact test for comparison of proportions. Multi-group comparisons were performed using ANOVA for normally distributed data, the Kruskal-Wallis test for continuous non-parametric data and the Chi-squared or Fisher’s exact test for comparison of proportions. Matched parametric data was compared using the paired *t*-test.

Birthweight analyses only included congenitally normal live born singleton infants with a weight measured within 24 h of delivery. Associations between AN, smoking, malaria, alcohol use, ethnic origin, residence, literacy level, income (participant and partner), gravidity, nutritional status, bed net use, height, frequency of antenatal clinic visits, receipt of IPTp, and infant gender with birthweight/LBW as well as anaemia at delivery (Hb <11 g/dL) were assessed. Covariates with *P* < 0.1 were included in a multivariate logistic regression model (LBW, anaemia) or linear regression (birthweight) as a starting model for a backward stepwise elimination model selection procedure and considered independent risk factors if *P* < 0.05. Tests for interaction between AN use and gravidity (categorised as primigravida, multigravida), height (categorised as low [<150 cm] and normal height [≥150 cm]), and participant’s ethnic grouping (categorized as highlander parentage and non-highlander parentage) were performed on the bivariate models for LBW and anaemia using the likelihood ratio test.

Multivariable analyses were conducted only amongst women with complete data (imputation was not performed). Background characteristics and chewing habits of women lost to follow-up were compared to those included in analyses to assess internal validity. External validity of the cohort is discussed elsewhere [29]. A formal sample size calculation for this study was not performed, as women were co-enrolled in a clinical trial that was powered to evaluate the effect of IPTp with SPAZ on LBW [29].

## Ethics

Ethical approval for this research was obtained from the PNG Institute of Medical Research Institutional Review Board (0815), the PNG Medical Research Advisory Council (08.01) and the Melbourne Health Human Research Ethics Committee (2008.162). The original trial was registered with the United States National Institutes of Health Clinical Trials Registry (NCT01136850). All participants provided informed written consent.

## Results

**Fig. 1:**
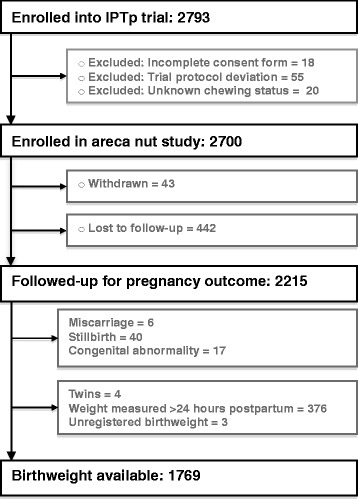
Participant flow diagram

A total of 2700 women were enrolled in the study (Fig. 1). Overall, 50.2 % (1354/2697) of women were primigravid, 62.6 % (1685/2691) resided in rural areas, 81.8 % (2107/2577) were anaemic, 89.2 % (2404/2694) were illiterate (defined as no education or less than grade 3) and 55.7 % (1428/2562) pursued an income-generating activity.

The prevalence of AN use was high (83.3 %; 2249/2700), with the largest number of women using 3–5 ANs per day. Baseline characteristics of AN users differed from non-users (Table 1). Tobacco smoking and alcohol use was reported by 18.9 % (511/2700) and 5.0 % (135/2688) of women, respectively, and both were more common among AN users (Table 1). Relatively few women of Highlands origin use AN, and non-users were less commonly anaemic at enrolment compared to users (Table 1). Heavy users (≥6 nuts/day) were less likely to be primigravid compared to light chewers and non-chewers (38.5 % [213/553] vs. 53.1 % [1141/2147], *P* <0.001) (Table 1).

**Table. 1:** Enrolment characteristics of pregnant women and association with areca nut use (*n* = 2700)

Characteristics	Non-users		Occasional users (<1/day)		Mild users (1-2/day)		Moderate users (3–5/day)		Heavy users (≥6/day)		P^a^	P^*b*^
Areca nut	16.7	(451)	11.5	(310)	24.1	(650)	27.3	(736)	20.5	(553)	-	-
Smoking	5.3	(24/451)	13.6	(42/310)	16.2	(105/650)	22.0	(162/736)	32.2	(178/553)	<0.001	<0.001
Alcohol	2.5	(11/444)	5.2	(16/309)	4.9	(32/649)	5.6	(41/735)	6.4	(35/551)	0.07	0.007
Rural residence	57.0	(257/451)	56.5	(175/310)	63.7	(414/650)	63.7	(469/736)	66.9	(370/553)	0.003	0.009
Highlands origin	12.2	(55/451)	8.4	(26/310)	4.6	(30/650)	4.8	(35/736)	4.0	(22/553)	<0.001	<0.001
Literate	89.6	(404/451)	90.9	(281/309)	89.4	(580/649)	89.9	(660/734)	86.9	(479/551)	0.36	0.80
Generates income	50.1	(221/441)	55.7	(165/296)	57.4	(347/605)	57.9	(402/694)	55.7	(293/526)	0.11	0.009
Anaemia (Hb < 11 g/dL)	73.4	(318/433)	82.7	(239/289)	81.9	(514/628)	84.1	(590/702)	85.0	(446/525)	<0.001	<0.001
Malaria^c^	6.7	(30/449)	10.0	(31/310)	8.3	(54/648)	7.0	(51/734)	5.3	(29/551)	0.09	0.62
Primigravidae	52.8	(238/451)	62.6	(194/310)	58.5	(380/650)	44.7	(329/736)	38.5	(213/553)	<0.001	0.22
MUAC <23 cm	26.3	(115/437)	17.1	(52/304)	31.3	(200/639)	30.0	(215/716)	27.1	(148/547)	<0.001	0.50
Age (years)	24.0	[20.0–28.0]	23.0	[20.0–26.0]	22.0	[19.0–26.0]	24.0	[20.0–28.0]	25.0	[21.0–29.0]	<0.001	0.22
Haemoglobin (g/dL)	9.9	[9.2–11.0]	9.8	[9.0–10.6]	9.6	[8.8–10.6]	9.6	[8.8–10.4]	9.5	[8.6–10.3]	<0.001	<0.001
Mean arterial pressure	78.0	[71.7–84.3]	77.7	[72.0–84.7]	78.0	[71.7–83.7]	77.0	[71.0–83.3]	76.0	[69.7–82.0]	0.004	0.16
Fundal height (cm)	22.0	[18.0–24.0]	22.0	[19.0–24.0]	22.0	[19.0–24.0]	22.0	[20.0–24.0]	22.8	[20.0–24.0]	<0.001	0.006

NOTE: Data are % (no. /total), or median [IQR]; p-values are for comparisons across the different groups of chewers. *MUAC* mid-upper arm circumference

^a^ *P*-values for comparison across all 5 groups. Bonferroni adjusted cut-off of significance: *P* = 0.005

^b^ *P*-values for comparison of users (occasional, mild, moderate, heavy) versus non-users

^c^By light microscopy, any *Plasmodium spp*

A total of 2215 women (82.0 % of enrolled) completed follow-up and had pregnancy outcome data recorded (Fig. 1). Women who were lost to follow-up (*n* = 485) were more likely to be primigravid (56.0 % [271/484] vs. 48.9 % [1083/2213], *P* = 0.005) but did not differ in their AN use compared to those women with known pregnancy outcomes (*P* >0.1 for the comparison of users against non-users and comparison across all 5 groups). Table 2 shows the associations between AN use and birth outcomes. There were no associations between AN use and stillbirth, miscarriage or prevalence of congenital abnormalities A total of 1769 women had infant weights that were eligible for inclusion in the birthweight analysis (Fig. 1). The overall prevalence of LBW was 13.9 % (245/1769). There were no statistically significant differences in the prevalence of LBW and PTD by AN chewing status (Table 2).

**Table 2:** Pregnancy and delivery outcomes according to areca nut use

Outcomes	Non-users		Occasional users (<1 nut/d)		Mild Users (1-2 nuts/d)		Moderate Users (3–5 nuts/d)		Heavy users (>5 nuts/d)		P^*a*^	P^b^
Miscarriage	0.5	(2/371)	0.0	(0/254)	0.6	(3/539)	0.2	(1/590)	0.0	(0/461)	0.34	0.27
Stillbirth	1.1	(4/371)	1.6	(4/254)	1.9	(10/539)	2.2	(13/590)	2.0	(9/461)	0.80	0.39
Congenital abnormality	1.4	(5/371)	0.4	(1/254)	0.6	(3/539)	0.5	(3/590)	1.1	(5/461)	0.52	0.19
Birthweight (g)	2966	[493]	2996	[507]	2928	[460]	2955	[468]	2971	[457]	0.49	0.76
LBW (<2,500 g)	14.5	(45/310)	13.8	(29/210)	15.1	(64/424)	12.1	(57/470)	14.1	(50/355)	0.76	0.71
Gestational age (exact weeks)	39.4	[1.6]	39.2	[1.5]	39.5	[1.6]	39.3	[1.9]	39.4	[1.6]	0.69	0.84
PTD (<37 weeks)	6.6	(14/212)	7.7	(11/143)	8.6	(21/245)	7.8	(23/308)	8.4	(20/239)	0.95	0.46
Male infant	39.1	(123/309)	44.0	(92/209)	44.2	(187/423)	48.2	(226/469)	46.6	(165/354)	0.21	0.045
Cord haemoglobin (Hb), g/dL	13.8	[2.3]	14.3	[2.4]	13.8	[2.6]	13.6	[2.5]	13.8	[2.3]	0.27	0.98
Hb, g/dL	10.5	[1.7]	10.3	[1.7]	10.1	[1.7]	9.9	[1.7]	10.0	[1.6]	<0.001	<0.001
Hb <11 g/dL	63.9	(182/285)	72.7	(141/194)	74.0	(279/377)	76.9	(326/424)	75.9	(242/319)	0.002	<0.001
MAP (mmHg)	85.5	[79.3–93.3]	87.3	[80.0–95.7]	86.6	[80.0–94.0]	84.0	[78.3–92.0]	84.7	[78.3–92.0]	0.003	0.41

NOTE: Data are % (n), mean [standard deviation], or median [interquartile range]. *LBW* low birthweight, *PTD* preterm delivery, *MAP*, mean arterial pressure

^a^ *P*-values for comparison across all 5 groups. Bonferroni adjusted *P*-value: 0.005

^b^ *P*- values for comparison of users (occasional, mild, moderate, heavy) versus non-users

Table 3 summarises associations of maternal and infant characteristics with birthweight. The mean difference in birthweight between non-users and AN users was −9 g (95 % CI −67, 49; *P* =0.76) (Table 3). Similarly, adjusted multivariate analyses did not demonstrate an effect of AN use on LBW or mean birthweight (Table 3). Smoking was not independently associated with an increased risk of LBW or reduced birthweight. There was no statistically significant difference between women who neither smoked or chewed, women who did not smoke but chewed, and women who both smoked and chewed in terms of LBW (14.7 % [43/292] vs. 13.2 % [149/1128] vs. 15.9 % [53/349], *P* = 0.58) or mean birthweight (2964 vs. 2964 vs. 2934 g, *P* = 0.58).

**Table 3:** Maternal and infant characteristics and association with birthweight (*n* = 1,769)

Factors		Low birthweight	P	Adjusted odds ratio (95 % CI)^e^	P	Birthweight (g)	∆	P	Adjusted ∆^e^	P
Areca nut	Yes	200/1459 (13.7)				2957				
	No	45/310 (14.5)	0.71	0.94 (065, 1.38)	0.77	2966	−9	0.76	−1	0.97
Smoking	Yes	53/349 (15.2)				2935				
	No	192/1420 (13.5)	0.42	-		2694	−29	0.30	-	
Malaria^a^	Yes	24/194 (12.4)				2923				
	No	221/1575 (14.0)	0.53	-		2963	−40	0.27	-	
Highlander^b^	Yes	6/111 (5.4)				3263				
	No	239/1,658 (14.1)	0.008	0.34 (0.14, 0.80)	0.013	2938	325	<0.001	311	<0.001
Primigravida	Yes	175/869 (20.1)				2844				
	No	70/900 (7.8)	<0.001	2.91 (2.13, 3.96)	<0.001	3048	−214	<0.001	−214	<0.001
Generating	Yes	148/1,215 (12.2)				2978				
income^c^	No	92/535 (17.2)	0.005	0.77 (0.57, 1.05)	0.09	2919	59	0.016	22	0.35
Bed net use^d^	Regular	160/1253 (12.8)				2961				
	Irregular	84/513 (16.4)	0.046	0.77 (0.56, 1.05)	0.10	2954	7	0.78	16	0.51
Height <150 cm	Yes	61/317 (19.2)				2827				
	No	180/1423 (12.7	0.002	1.71 (1.22, 2.41)	0.002	2987	−160	<0.001	−151	<0.001
MUAC <23 cm	Yes	85/471 (18.1)				2861				
	No	155/1260 (12.3)	0.002	1.49 (1.10, 2.03)	0.010	2994	−133	<0.001	−99	<0.001
Antenatal visits	1-2	60/316 (19.0)				2889				
	≥3	185/1453 (12.7)	0.004	0.65 (0.46, 0.92)	0.014	2973	−84	0.004	−73	0.010
SPAZ-IPTp	Yes	107/892 (12.0)				2974				
	No	138/877 (15.7)	0.023	0.64 (0.48, 0.85)	0.003	2942	32	0.15	44	0.041
Female infant	Yes	158/971 (16.3)				2925				
	No	86/793 (10.8)	0.001	1.64 (1.22, 2.22)	0.001	2999	−74	0.001	−78	<0.001

Note: Data are n (%). *CI* confidence interval, *MUAC* mid-upper arm circumference

^a^Peripheral parasitaemia at any stage during pregnancy, by light microscopy

^b^Both maternal parents are from highlands region

^c^Partner

^d^Insecticide-treated and untreated nets combined, irregular users include 9 non-users

^e^Model includes 1680 observations

Women using AN were more likely to be anaemic at delivery on crude (OR [95 % CI]: 1.66 [1.72, 2.25], *P* < 0.001, *n* = 1599) and adjusted analysis (OR [95 % CI]: 1.67 [1.27, 2.20]) (Table 4). Amongst women with complete data for haemoglobin and with a dating ultrasound (*n* = 1053) gestational age at delivery was not associated with haemoglobin levels (*P* = 0.17), and when included in the final multivariate model the association between AN use and anaemia at delivery remained significant (OR [95 % CI]: 1.87 [1.35, 2.59], *P* < 0.001, *n* = 1053). There was an interaction between gravidity and AN use on anaemia at delivery (*P* = 0.027): the negative impact of chewing on anaemia appeared more profound amongst multigravidae (chewers: 78.1 % [523/670] vs. non-chewers: 59.5 % [78/131], *P* < 0.001) compared to primigravidae (chewers: 72.2 % [465/644] vs. non-chewers: 67.5 % [104/154], *P* =0.25).

**Table 4:** Maternal characteristics and anaemia (haemoglobin <11 g/dL) at delivery (*n* = 1,599)

Factors		Anaemia	Odds ratio (95 % CI)	P	Adjusted OR^c^ (95 % CI)	P
Areca nut	Yes	988/1314 (75.2)				
	No	182/285 (63.9)	1.72 (1.31, 2.25)	<0.001	1.67 (1.27, 2.20)	<0.001
Smoking	Yes	234/315 (74.3)				
	No	936/1284 (72.9)	1.07 (0.81, 1.42)	0.62	-	
Malaria^a^	Yes	141/178 (79.2)				
	No	1029/1421 (72.4)	1.45 (0.99, 2.12)	0.06	1.45 (0.99, 2.12)	0.06
Highlander^b^	Yes	67/103 (65.1)				
	No	1103/1496 (73.7)	0.66 (0.44, 1.01)	0.06	0.74 (0.48, 1.14)	0.17
Primigravida	Yes	569/798 (71.3)				
	No	601/801 (75.3)	0.83 (0.66, 1.03)	0.08	0.85 (0.67, 1.04)	0.11
Height <150 cm	Yes	201/289 (69.6)				
	No	952/1286 (74.0)	0.80 (0.61, 1.06)	0.12	-	
MUAC <23 cm	Yes	316/420 (75.2)				
	No	824/1143 (72.1)	1.18 (0.91, 1.52)	0.22	-	
Generates income	Yes	617/829 (74.4)				
	No	493/692 (71.2)	1.17 (0.94, 1.47)	0.16	-	
≥3 antenatal visits	Yes	950/1310 (75.5)				
	No	220/289 (76.1)	0.82 (0.62, 1.11)	0.21	-	
SPAZ-IPTp	Yes	583/800 (72.9)				
	No	587/799 (73.5)	0.97 (0.78, 1.21)	0.79	-	

Note: Data are n (%). *CI* confidence interval. *MUAC* mid-upper arm circumference

^a^Peripheral parasitaemia during pregnancy, by light microscopy

^b^Both maternal parents from highlands region

^c^Model includes 1596 number of observations

Amongst women who had data for haemoglobin levels at both enrolment and delivery (*n* = 1534) mean Hb levels were higher at delivery compared to enrolment (10.1 vs. 9.8 g/dL, *P* < 0.001). However, the magnitude of mean change in Hb levels between the two time points did not differ between chewers and non-chewers in crude analysis (0.38 vs. 0.38 g/dL, *P* > 0.99) or in an analysis that adjusted for time difference between measurements (*P* = 0.71).

## Discussion

Our research represents the second largest study of the impact of AN chewing in pregnancy on birth outcomes, and is the most comprehensive for PNG. In a cohort of 2,700 pregnant women with a high proportion of AN users (83.3 %), most of whom were regular users (86.2 %), chewing AN was associated with anaemia at enrolment and delivery but not with adverse pregnancy outcomes such as LBW and stillbirth. The prevalence of gestational AN use was comparable to that observed previously in pregnant women in coastal PNG [15, 20, 35], and is similar to use amongst the general population [13, 28].

The majority of studies that have previously evaluated the effect of AN consumption on the health of mother and baby originate from PNG and Taiwan [15, 16, 20–22]. They have reported significant associations between maternal AN chewing and reduction in mean birthweight [15, 16, 20], lower male newborn rate, lower birth length and higher prevalence of LBW [16] and increased risks of pregnancy loss [21, 22]. Observations from these earlier studies stand in contrast to findings from this research and those of the largest population study of AN use in pregnancy conducted to date (Thailand) [17], neither of which demonstrated an association between AN and adverse pregnancy outcomes such as LBW and pregnancy loss. This comes in spite of significant differences in chewing practices and participant characteristics between cohort studies with regards to AN consumption and smoking. First, intensity of AN consumption differed: most women in the Thai study were occasional/light AN users, whilst most PNG women were regular, if not heavy users. Second, the prevalence of smoking differed considerably. Smoking was more common in the Thai cohort (~38 % vs. 19 % in PNG) where it reduced mean birthweight, whilst the habit was not associated with lower mean birthweight in the present study (−29 g, *P* = 0.30).

Observations from our study and Thailand suggest that the deleterious effect of AN use on pregnancy outcomes is perhaps less pronounced at the population level than previously suggested. Larger population cohort studies have more power to detect true associations and are less prone to selection bias (e.g. preferential recruitment of heavy chewers that may have concomitant risk factors of adverse birth outcomes) [36] which may partly explain why more recent, larger cohort studies on AN use in pregnancy did not replicate the findings of previous, smaller, studies. Furthermore, previous research did not control for confounders such as parity [16], malaria and maternal undernutrition [15, 20] and substance abuse [16, 21]. Earlier studies from PNG were conducted prior to intensified malaria control, and malaria in pregnancy was common [37] whereas malaria prevalence was comparatively low in this cohort and hence less likely to be a potential confounder of the AN-birthweight relationship. Additionally, AN use may largely be associated with adverse pregnancy outcomes only amongst women with pre-existing health problems or presence of risk factors, an observation which could be missed by large cohort studies. Population-based studies and studies identifying at-risk women, as well as research evaluating the physiological effects of chewing on e.g. utero-placental blood flow, will assist with broadening our understanding of the effects of AN use in pregnancy and may help consolidate the findings of recent large cohort studies.

A novel observation in this study was the association of AN use with anaemia at enrolment and delivery, which persisted after adjusting for other factors associated with haemoglobin levels including malaria, gravidity, socioeconomic factors, maternal undernutrition and ethnicity. This is important as anaemia has been associated with adverse pregnancy outcomes [38], increases the risk of heart failure, and reduces a woman's reservoir to withstand the consequence of postpartum haemorrhage. In Thailand, AN use was associated with anaemia at delivery on crude, but not adjusted analyses. Heterogeneity in participant characteristics and chewing intensity between these studies may be one explanation. It is also possible that we did not measure and adjust for all relevant confounders of the AN-anaemia relationship. Assuming that the association between AN chewing and anaemia is real and confirmed in future studies, potential mechanisms as to how AN use affects haemoglobin remain to be elucidated. Arecoline has been shown to modify appetite [39] which may result in a global reduction of food intake including essential nutrients such as iron, thereby increasing the risk of iron-deficiency anaemia. AN has also been associated with irritation of the lower oesophagus and gastritis [40] which may result in chronic blood loss and iron-deficiency anaemia. Furthermore, components of the betel nut quid may, if swallowed, alter the ability of the gastrointestinal tract to absorb nutrients such as iron. Further research into potential mechanisms may be warranted, and pregnant women with anaemia should receive advice about stopping chewing of AN. The effect of chewing on Hb levels was most pronounced amongst multigravidae, which may be explained by the fact these women are more likely to be heavy users and are likely to have been chewing AN for longer (chronic exposure). Finally, there was no statistically significant difference in the change in Hb levels from enrolment until delivery by chewing status. This suggests that it is the effect of AN use on haemoglobin levels before pregnancy that by and large dictates the association between AN and anaemia at delivery.

We observed a higher proportion of male infants amongst AN users compared to non-users. This is not the first time such an association was observed, although previous research suggested a reduction in the male-to-female sex ratio amongst chewers [16]. Heavy cigarette smoking has also been shown to affect the sex ratio at birth [41]. Potential mechanisms that could explain such observations include differential effects of AN on spermatozoae (but we did not document AN use of partner) or embryo survival [22], although most AN studies, including ours, did not observe an association of AN use with miscarriage and stillbirth.

Strengths of this study include a large cohort size, detailed information on chewing habits and participant characteristics, and a comprehensive assessment of the effect of AN use on a number of adverse pregnancy outcomes and maternal anaemia. Our study has limitations. Firstly, AN use was only assessed at first antenatal visit, and chewing habits may have changed through the course of pregnancy, which may introduce bias. Second, AN use was self-reported and was not corroborated by arecoline blood levels. Third, we did not evaluate quantity and type of tobacco use. It is plausible that smoking was not associated with birthweight in this study, and another study from the same area [15], because of infrequent consumption or type of tobacco used. However, of concern is that proportion of women smoking in pregnancy appears to have doubled within five years (9 % vs. 18 %) [15]. Fourth, we may not have measured all factors associated with our outcome measures (e.g. indoor pollution, iron/folate supplementation and adherence), and residual confounding may explain some of our findings. Furthermore, gestational length could only be adequately assessed (by ultrasound) in a subset of women, increasing the risk of selection bias and requiring cautious interpretation of these data. Lastly, we did not perform detailed full blood counts and measurements of iron and folate concentrations, precluding a more detailed characterisation of anaemia in our cohort.

Although the present study was unable to demonstrate an association between AN chewing and adverse birth outcomes, we observed an increased risk of anaemia amongst chewers. Further evaluation of this association is required. Irrespective of the apparent absence of harmful effects of AN on pregnancy shown in this study, the relationship between chronic exposure to AN and other serious health problems (cancer in particular) has been well established and this calls for strategies to reduce AN use. In settings where resources are limited, antenatal care should focus on, and prioritize interventions that target other known and preventable causes of adverse pregnancy outcomes such as malaria and tobacco smoking, whilst raising awareness of the known adverse effects of AN.

## Conclusions

The present study was unable to demonstrate significant associations between AN use in pregnancy and reduced birthweight and pregnancy loss. However, AN use was associated with lower haemoglobin levels at first antenatal visit and delivery, and appeared to affect the male-to-female sex ratio. Gestational AN use should be discouraged, given the potential adverse effects on haemoglobin and well-established long-term health risk including oral cancer. Future research evaluating the potential association of AN use and anaemia may be warranted.

## Abbreviations

AN: *Areca catechu* nut
ANOVA: Analysis of variance
CI: Confidence interval
Hb: Haemoglobin
HIV: Human immunodeficiency virus
IPTp: Intermittent preventive treatment in pregnancy
IQR: Interquartile range
LBW: Low birthweight
MAP: Mean arterial pressure
MUAC: Mid-upper arm circumference
OR: Odds ratio
PNG: Papua New Guinea
PTD: Preterm delivery
SD: Standard deviation
SPAZ: Sulphadoxine-pyrimethamine plus azithromycin
